# Food Authentication: Identification and Quantitation of Different Tuber Species via Capillary Gel Electrophoresis and Real-Time PCR

**DOI:** 10.3390/foods9040501

**Published:** 2020-04-16

**Authors:** Stefanie Schelm, Melanie Siemt, Janin Pfeiffer, Christina Lang, Hans-Volker Tichy, Markus Fischer

**Affiliations:** 1 Hamburg School of Food Science, Institute of Food Chemistry, University of Hamburg, Grindelallee 117, 20146 Hamburg, Germany; stefanie.schelm@chemie.uni-hamburg.de (S.S.); melanie.siemt@studium.uni-hamburg.de (M.S.); janin.pfeiffer@studium.uni-hamburg.de (J.P.); christina.lang@chemie.uni-hamburg.de (C.L.); 2 LUFA-ITL GmbH, Dr.-Hell-Straße 6, 24107 Kiel, Germany; hans-volker.tichy@agrolab.de

**Keywords:** truffle, *T. melanosporum*, *T. indicum*, real-time PCR, RFLP, quantitative evaluation

## Abstract

Truffles are hypogeous fungi mainly found in Europe and Asia. Due to their special aroma and taste, some truffle species are sold on the international market at an extremely high price. Among the economically relevant species, the white Alba truffle (*Tuber magnatum*) and the black Périgord truffle (*T. melanosporum*) are the most appreciated species. The fruiting bodies of the Asian black truffle are morphologically very similar to *T. melanosporum,* and those of the Bianchetto truffle (*T. albidum* Pico) are similar to *T. magnatum*, but are of little economic value. Highly valued species are adulterated with cheaper ones, especially. Because of this problem, the aim of this study was the development of methods for detecting possible admixtures to protect consumers from fraud. This study is based on seven different truffle species (117 fruiting bodies) from different growing regions. Additionally, selected truffle products were included. Using this material, a real-time PCR (polymerase chain reaction) assay allowing the detection and quantitation of Asian black truffles in *T. melanosporum* up to 0.5% was developed. In addition, a capillary gel electrophoresis assay was designed, which allows the identification and quantitation of different species. The methods can be used to ensure the integrity of truffle products.

## 1. Introduction

Truffles are underground fungi belonging to the class of the Ascomycetes in the order Pezizales [1,2]. They grow in an ectomycorrhizal symbiosis with roots of different trees and shrubs, e.g., oak, poplar, willow, hazel [3], and Cistus [4]. Tuber spp. are mainly distributed in Europe, Asia, North Africa, and America [5,6]. At least 180 Tuber species exist worldwide [6], 70–75 species have been well described [7], and 32 species are currently listed in Europe [8].

Under specific environmental conditions, such as calcareous soil with a neutral pH [9], truffles produce hypogeous edible ascocarps. The unique aroma and taste emitted from the fruiting bodies are responsible for the gastronomical desirability; therefore, some truffles represent some of the most highly prized edible and valuable mushrooms worldwide [10]. 

*T. magnatum* is the most expensive truffle species in general [6]. It is mainly distributed in Italy, but it can also be found in the area around Balkan [11], France, and Switzerland [6]. Another white (or whitish) truffle with lesser economic value, *T. albidum* Pico, is morphologically and biochemically similar to *T. magnatum*, which can be subject to fraud [12]. It is also possible that roots initially colonized by *T. magnatum* have produced other white truffles, such as *T. albidum* Pico [5].

Among the black truffles is the Périgord truffle *T. melanosporum,* the most expensive species which is highly valued for its organoleptic properties [13], and, therefore, there is a risk of fraud. The natural distribution area is mainly France, Spain, and Italy [14]. The Asian black truffles, such as *T. indicum* and *T. himalayense,* are closely related to *T. melanosporum,* and the fruiting bodies are morphologically very similar [15]. Because of the larger production value, *T. indicum* is sold at a lower price and imported from China to Europe, North America, and Australia [16,17,18]. Cases have been reported where *T. indicum* has been sold as *T. melanosporum,* and incorrect inoculations and incidence of ectomycorrhiza from *T. indicum* in *T. melanosporum* truffle orchards have been found [16,18,19,20]. Due to the lower price, admixture from the Asian black truffles with *T. melanosporum* is sometimes observed in food products. Since the microscopic identification of truffle fruiting bodies is difficult, molecular methods have been introduced to analyze different truffle species that are morphologically similar.

One region of the DNA suited for the molecular analysis of fungi is the rDNA (ribosomal DNA), which contains two variable non-coding regions, the internal transcribed spacer (ITS) region 1 and 2, between the highly conserved 17S, 5.8S and 25S rRNA (ribosomal RNA) genes [21]. The ITS regions are widely used to analyze ectomycorrhizal communities of mycorrhizal fungi and fungal species in the field, and it is recommended to be used as the primary fungal barcode [22,23]. Another advantage of the ITS region is the repetitive character resulting in a low detection limit [24,25,26].

Molecular methods based on the ITS region have also been widely used for the identification of truffle species [27,28,29,30,31,32]. Methods targeting the rDNA region for detecting admixtures from lower prized truffle species in *T. melanosporum* were developed, enabling the qualitative detection of ectomycorrhiza or ascocarps from *T. indicum* in *T. melanosporum* [20,30,33,34]. Different real-time PCR (polymerase chain reaction) methods for truffles were developed, e.g., for the analysis of truffle grounds and the quantitation of mycelium in soil [35,36,37,38]. Furthermore, real-time PCR assays for the detection of *T. melanosporum* in processed food products and for the quantitation of *T. aestivum* in mycelium have been developed [35,39,40]. To our knowledge, there is currently no real-time-PCR method available, which can quantify Asian truffles in *T. melanosporum*.

To detect possible admixtures of cheaper truffle species and to protect consumers from fraud, the aim of this study was to develop methods to detect such possible admixtures. The DNA-based methods can be used for quality control in the food industry or in official food control to ensure the integrity of truffle products. 

The present paper reports the application of molecular techniques, real-time PCR, capillary gel electrophoresis (CGE), and restriction fragment length polymorphism (RFLP) to identify and quantify admixtures of different truffle species. A specific primer pair (with minor modifications) for *T. indicum* [33] and a new *T. melanosporum* specific primer pair suitable for real-time PCR with hybridization probes were used. The real-time PCR technology with hybridization probes was chosen for the real-time PCR assay. Compared to assays with SYBR Green I, hybridization probes are more specific because the fluorescent signal is derived from a specific probe and thus, is sequence-specific [41]. Moreover, a quantitative CGE based method for species differentiation and a RFLP assay combined with CGE were developed. The RFLP offers an alternative to real-time PCR as an easy to use method. The methods developed were tested on fruit bodies and truffle products from retail outlets.

## 2. Materials and Methods

### 2.1. Sample Material

In total, 117 fruiting bodies of different truffle species from distinct origins were analyzed (see Table 1). Upon arrival, all fruiting bodies were frozen in liquid nitrogen and stored at –80 °C. Furthermore, canned truffle fruiting bodies and food products containing truffles purchased at retail locations were used.

### 2.2. DNA Isolation

For DNA isolation of the matrix mixtures, commercially available kits (QIAGEN DNeasy^®^ Plant Mini Kit (QIAGEN, Hilden, Germany), peqGOLD Fungal DNA Mini Kit (VWR International GmbH, Darmstadt, Germany)) were used. DNA purity was determined photometrically using a DS-11 Spectrophotometer (DeNovix Inc., Wilmington, USA). DNA concentration was determined fluorometrically (Quantus^TM^ Fluorometer, Promega GmbH, Mannheim, Germany).

For a high sample throughput, the simple “alkaline” and the “modified PCI (phenol-chloroform-iso-amyl alcohol)” DNA extraction method, originally developed for tissue samples of chicken embryos [42], were used with slight modifications. In the “alkaline method”, approximately 25 mg of sample material was incubated for 20 min at 75 °C in 100 µL 0.2 M NaOH after grinding with a micropistille in a 1.5 mL reaction tube. Afterward, 300 µL 0.04 M Tris/HCl was added. One microliter of the liquid phase was used directly for PCR. Additionally, the “modified PCI method” was used as followed: Approximately 25 mg sample material was ground in 500 µL extraction buffer (0.1 M Tris/HCl, 55 mM CTAB, 1.4 M NaCl, and 20 mM EDTA, pH 8.0) [43] with a micropistille in a 1.5 mL reaction tube and incubated for 30 min at 65 °C. Five hundred microliters chloroform were added and centrifuged at 10,000 × *g* for 5 min. The supernatant was transferred to a new 2 mL reaction tube and mixed with 500 µL isopropanol and incubated for 30 min at 4 °C. After repeated centrifugation for 15 min, the supernatant was discarded, and the pellet was washed with 500 μL 70% ethanol. The DNA pellet was vacuum-dried and dissolved in 50 μL water. One microliter was used directly for PCR.

### 2.3. Preparation of Spiked Sample Material

#### 2.3.1. DNA Mixtures

The DNA isolated from different truffle fruiting bodies was adjusted to a concentration of 5 ng/µL and mixed in different ratios (0.1%, 0.5%, 1%, 5%, 10%, 20%, 40%, 70% DNA isolated from *T. indicum* in DNA isolated from *T. melanosporum*; 5%, 20%, 40%, 80% DNA isolated from *T. albidum* Pico in DNA isolated from *T. magnatum*). 

Additionally, mixtures of PCR amplicons were prepared by mixing PCR products from different fruiting bodies in different ratios after PCR (20%, 40%, 50% *T. indicum* in *T. melanosporum* PCR amplicons; 5%, 20%, 40%, 80% *T. indicum* in *T. aestivum* and *T. albidum* Pico in *T. magnatum* PCR amplicons).

#### 2.3.2. Matrix Mixtures of Fruiting Bodies

Spiked samples of two distinct truffle species were produced by weighing out ground fruiting bodies of different truffle species in a 2 mL reaction tube (4.3%, 4.6%, 7.4%, 13.5%, 18.28%, 20.4%, 32.2% and 11.2%, 21.7%, 28.3%, 47.5% *T. indicum* in *T. melanosporum*). The ground powder was mixed in 500 µL DNA isolation buffer using a bead ruptor (Bead Ruptor 24; Biolabproducts GmbH, Bebensee, Germany) and the DNA isolation protocol was continued.

### 2.4. Real-Time PCR

To detect and quantify possible contamination with lower-priced Asian truffles of the *T. indicum* complex (*T. indicum*/*himalayense*) in higher priced truffles, such as *T. melanosporum,* a specific primer pair (with minor modifications) designed from Paolocci et al. (1997) [32] was used (Indi-fw/ITS4LNG, see Table 2). This primer pair targets the ITS2 region on the rDNA. Additionally, a primer pair specific for *T. melanosporum* (Mela-fw/Mela-rv, see Table 2), also located in the ITS2 region, was designed using the sequences from Paolocci et al. (1997) [32] as templates, which was able to detect the presence of *T. melanosporum*. 

For real-time PCR assays, hybridization probes labeled with a fluorophore (Hex, Cy5) and a quencher (BHQ2) were designed, taking care that no overlapping of fluorescence maxima occurred. All primer and probe sequences, including the fluorophores and quenchers, used in this work are listed in Table 2.

The real-time PCR assay was performed in a volume of 10 μL including 1× Taq reaction buffer (Biozym Scientific GmbH, Hessisch Oldendorf, Germany), 0.8 mM dNTPs (each 2.5 mM, Bioline GmbH, Luckenwalde, Germany), 0.5 U Taq polymerase (Biozym Taq DNA Polymerase, Biozym Scientific GmbH, Hessisch Oldendorf, Germany), 50 nM of each primer (Life Technologies, Darmstadt, Germany), 40 nM of the fluorescently labeled probe (Eurofins Genomics GmbH, Ebersberg, Germany), and 1 µL of isolated DNA. 

For real-time PCR, a CFX96 Touch System thermocycler (Bio-Rad Laboratories GmbH, Munich, Germany) was used. Real-time PCR was performed with the following two-step temperature program: initial denaturation for 300 s at 95 °C followed by 30 cycles with 20 s denaturation at 95 °C and 60 s annealing and elongation at 60 °C, finished by final elongation for 600 s at 72 °C. 

#### Practical Determination of LoD

To determine the LoD (limit of detection) of the developed real-time PCR DNA, mixtures of *T. melanosporum* and *T. indicum* (0.1%, 0.5%, 1%, 5%, 10%, 20%, 40%, 70% DNA isolated from *T. indicum* in DNA isolated from *T. melanosporum*) were measured in duplicate using the primer pair specific for *T. indicum* with 10 ng DNA in each PCR reaction. 

### 2.5. Isolation of DNA Fragments from Agarose Gels

For the isolation of PCR fragments from agarose gels, the peqGOLD^®^ Gel Extraction Kit (VWR International GmbH, Erlangen, Germany) was used according to the manufacturer’s information.

### 2.6. RFLP and CGE

For the amplification reactions, the primers ITS1 and ITS4 [21] amplifying the ITS region were used. The PCR prior to the RFLP was performed in a volume of 10 μL including 1× Taq reaction buffer (Biozym Scientific GmbH, Hessisch Oldendorf, Germany), 0.8 mM dNTPs (each 2.5 mM, Bioline GmbH, Luckenwalde, Germany), 0.5 U Taq polymerase (Biozym Taq DNA Polymerase, Biozym Scientific GmbH, Hessisch Oldendorf, Germany), 1 μM of each primer (Life Technologies, Darmstadt, Germany), and 1 µL of isolated DNA. The thermal cycle profile was as follows: initial denaturation for 300 s at 95 °C, 35 cycles with denaturation for 20 s at 95 °C, annealing for 20 s at 47.3 °C, elongation for 20 s at 72 °C, and final elongation for 300 s at 72 °C. PCR amplicons were visualized with agarose gel electrophoresis (AGE) on 1.5% agarose gels stained with 0.001% ethidium bromide. The gels were visualized under UV light (254 nm, Biostep, Felix 1040, Biostep GmbH, Jahnsdorf, Germany).

The restriction enzyme *Cvi*QI (Thermo Fisher Scientific Inc., Waltham, United States; restriction sequence: G/TAC) was used for restriction. To carry out the reaction, 1 U of restriction enzyme, 2 μL of PCR products, 0.8 µL corresponding buffer in a total volume of 8 µL were used. The reaction mixture was incubated at 25 °C for approximately 8 h without heat inactivation.

Detection of PCR products was carried out by AGE (see above). Quantitation by capillary gel electrophoresis was performed according to the manufacturer instructions on a 2100 Bioanalyzer (Agilent Technologies, Santa Clara, United States) using the Agilent DNA 7500 Kit (Agilent Technologies, Santa Clara, CA, United States) and on a Fragment Analyzer^TM^ (Advanced Analytical Technologies, Inc, Ankeny, IA, United States).

## 3. Results and Discussion

### 3.1. Real-Time PCR

#### 3.1.1. Primer Specificity

For the detection and quantitation of potential impurities of Asian black truffles in *T. melanosporum* a specific primer pair (with minor modifications) designed from Paolocci et al. (1997) [32] specific for these species was used in combination with a hybridization probe. In addition, a hybridization probe and a primer pair specific for *T. melanosporum* were designed to check the presence of this high prized truffle in the samples. The *T. melanosporum* specific primer pair was designed so that the length for the amplification product was about 140 bp. So the amplification length was similar to the amplification product of the *T. indicum* specific primer pair, and it meets the requirements for the hybridization probes [44] and allows an analysis of fragmented DNA as it could occur in processed food [24]. 

The specificity of the used primer was tested with DNA isolated from all samples of different *Tuber* spp. listed in Table 1. As can be seen in Table S1, the specific primer pair for *T. indicum* showed only positive PCR results with the Asian black truffles *T. indicum* and *T. himalayense*. Cross contaminations can be ruled out because none of the samples from other *Tuber* species showed positive Cq values. This demonstrates the suitability of the real-time assay for the detection and possible quantitation of *T. indicum/himalayense* and *T. melanosporum* without cross amplifications. This opens up the possibility to use this primer pair to detect possible admixtures of the cheaper Asian black truffles in higher-priced species, such as *T. melanosporum*. A similar performance was observed for the *T. melanosporum* specific primer, which gave only positive signals with the analyzed DNA isolated from *T. melanosporum*. In addition, the *T. melanosporum* fruiting bodies canned in saltwater from food retail showed positive Cq values in real-time PCR, showing that the real-time PCR assay also works with processed food. The ranging Cq values for analyzed fruiting bodies can be explained by the DNA isolation method used (“alkaline method”, “modified PCI method” [42]) because the concentration of DNA was not adjusted to a uniform level for specificity test. Since the specificity of the primer pairs should be tested qualitatively, the non-adjusted DNA concentration did not affect the specificity test negatively.

#### 3.1.2. Quantitation

**Figure 1:**
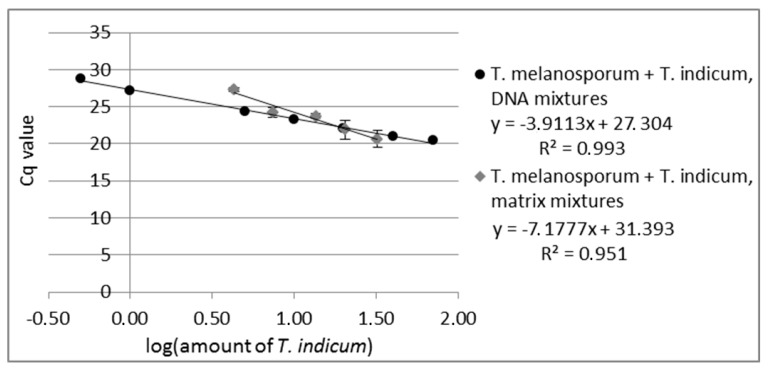
Standard curve of real-time PCR of DNA-mixtures from *T. melanosporum* with *T. indicum* with 0.5%, 1%, 5%, 10%, 20%, 40%, 70% *T. indicum* DNA and standard curve of matrix-mixtures from *T. melanosporum* with *T. indicum* with 4.3%, 7.4%, 13.5%, 20.4, 32.2% *T. indicum*. Each DNA-mixture was analyzed in duplicate and each matrix mixture in triplicate to real-time PCR with the primer pair specific to *T. indicum*. The Cq values are plotted against the logarithm of *T. indicum* amount.

Primer suitability for quantitation was tested by measuring DNA mixtures over the concentration range from 0.1% to 70% *T. indicum* in *T. melanosporum* DNA. The real-time assay of the DNA mixtures showed a reliable amplification over the concentration range from 0.5% to 70% *T. indicum* in *T. melanosporum* DNA (see Figure 1, measuring values are shown in Table S2) with an R^2^ of 0.993 (Equitation for *R*^2^ see Equitation S1). Due to the fact that the last measured standard (0.1% *T. indicum* in *T. melanosporum* DNA) gave no measurable signal, the LoD of the real-time PCR assay was set at 0.5% *T. indicum* in *T. melanosporum* for the *T. indicum* specific real-time assay. 

PCR can be influenced by the sample matrix [45], e.g., by coextracted substances. It is also possible that the DNA from some truffle species can be more easily isolated or that the DNA from some species contains more PCR inhibitors. This would lead to an inhomogeneous PCR amplification by samples with more than one truffle species. To assess these effects, five different matrix mixtures of *T. indicum* with *T. melanosporum* were prepared (4.3%, 7.4%, 13.5%, 20.4%, 32.2% *T. indicum*). For the matrix experiments, DNA from each matrix mixture was isolated and analyzed via real-time PCR with both real-time systems, the *T. indicum* and the *T. melanosporum* specific primers, in triplicate with 10 ng DNA pro PCR reaction. As in the case of the analyzed DNA mixtures, the standard curve of the matrix mixtures revealed a linear correlation between the Cq values plotted against the logarithm of *T. indicum* content in *T. melanosporum* with an R^2^ of 0.951 (see Figure 1). Eventually, coextracted PCR inhibitors could lead to PCR efficiency under 100%. The results obtained show that the developed real-time PCR opens the possibility to quantify the content of *T. indicum* admixtures in *T. melanosporum* also in matrix mixtures, to determine the rate of fraud by replacing expensive truffles by cheaper ones. 

Furthermore, two matrix mixtures of *T. melanosporum* fruiting bodies with different amounts of *T. indicum* (MM1: 18.28%, MM2: 4.60% *T. indicum*) were analyzed in duplicate via real-time PCR with *T. melanosporum* and *T. indicum* specific primer pair. Calculation of the *T. indicum* content was performed using absolute quantitation with an external calibration curve of DNA mixtures (0.1% to 70% *T. indicum* in *T. melanosporum* DNA) and with the external calibration curve of matrix mixtures used above. Using the calibration curve of DNA mixtures for MM1 and MM2, a *T. indicum* amount of 19.44% ± 6.4% or 2.32% ± 0.7%, respectively, was calculated, and using the calibration curve of matrix mixtures a *T. indicum* amount of 17.97% ± 3.16% (MM1) or 5.84% ± 0.92% (MM2) was calculated. The results show that the use of the matrix calibration curve leads to an improvement in quantitative results by compensating matrix effects. These results indicate that it should also be possible to determine the *T. indicum* content in food products or other matrices using a standard curve with a matrix comparable to the sample.

### 3.2. RFLP and CGE

#### 3.2.1. PCR-Amplification of the ITS Region, Evaluation via CGE

The primers ITS1/ITS4 amplify the ITS1, 5.8S, and ITS2 regions. PCR amplification of DNA with this primer pair from the different *Tuber* spp. resulted in bands on agarose gels with different lengths. *T. himalayense, T. indicum*, *T. melanosporum,* and *T. magnatum* generated bands with approximately 630 bp. Amplicons from *T. aestivum* DNA were approximately 50 bp longer. *T. albidum* Pico DNA resulted in bands on agarose gels with about 550 bp, and *T. brumale* DNA resulted in bands with approximately 900 bp (see Figures S1–S6). In contrast to the findings of Paolocci et al. (1995), the analyzed *T. aestivum* samples used in this study showed only one band on agarose gels with approximately 700 bp, which was also shown in, e.g., [46]. The fact that *T. brumale* showed the longest and *T. albidum* Pico the shortest amplicon length (900 bp and 500 bp, respectively) was also detected by other groups [47,48].

Due to the fact that *T. aestivum*, *T. albidum* Pico, and *T. brumale* showed fragments different in size compared with the other truffle species analyzed, an identification and quantitation of these species or another truffle species mixed with *T. aestivum*, *T. albidum* Pico and *T. brumale* should be possible via CGE.

To prove whether the detection and quantitation of *T. indicum* in a mixture with *T. aestivum* or *T. albidum* Pico mixed with *T. magnatum* based on the different amplicon length is possible, different mixtures of PCR amplicons produced with ITS1/ITS4 primers were prepared. Mixtures from *T. indicum* with *T. aestivum* and from *T. magnatum* with *T. albidum* Pico with 5%, 20%, 40%, and 80% *T. indicum/albidum* Pico PCR amplicons were analyzed on CGE and the relative peak area from *T. indicum* and *T. albidum* Pico was integrated. Additionally, isolated DNA of *T. albidum* Pico and *T. magnatum* were mixed prior to PCR to check if the PCR had an influence on the quantitation via the different ITS amplicon length on CGE.

**Figure 2:**
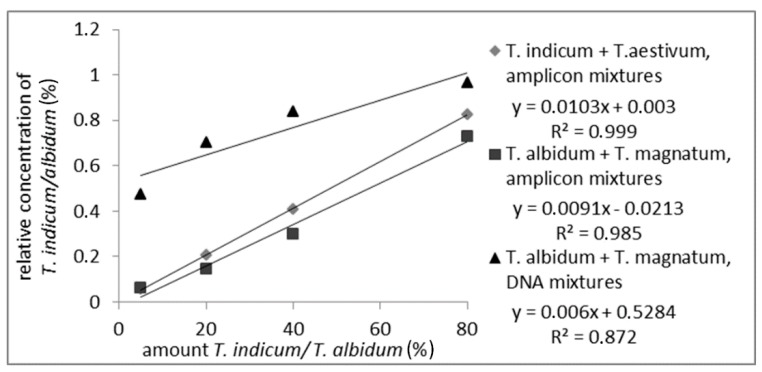
Standard curve of PCR-amplicon mixtures from *T. indicum* with *T. aestivum* and *T. albidum* Pico with *T. magnatum* with 5%, 20%, 40%, 80% *T. indicum/albidum* Pico and standard curve of DNA mixtures from *T. albidum* Pico with *T. magnatum* with 5%, 20%, 40%, 80% *T. albidum* Pico. The detected relative areas of PCR-amplicons are plotted against *T. indicum* or *T. albidum* Pico amount.

As shown in Figure 2 (measuring values are shown in Table S3), a correlation between the relative peak-area of the measured PCR-amplicons from *T. indicum* or *T. albidum* Pico and the amount of the corresponding truffle species could be detected over the whole range of analyzed samples with an R^2^ of 0.999 or 0.985, respectively. The R^2^ of 0.872 of the DNA mixture was lower than the R^2^ of the amplicon mixtures, but a linear correlation was still visible. The decline in the R^2^ can be explained by inhomogeneous samples, or matrix effect occurred during PCR. These results show that a quantitation of a truffle species mixed with another species is possible via just the different lengths of the PCR-amplicons, which makes the analysis fast and simple because no digestion with restriction enzymes is necessary.

#### 3.2.2. RFLP of the ITS Region, Evaluation via CGE

Due to the same length of the region amplified with the ITS1/ITS4 primers, a differentiation of the highly prized black Périgord truffle *T. melanosporum* and the Asian black truffles is not possible by comparing the ITS amplicon length. Thus, a differentiation and quantitation of admixtures from Asian black truffles of the *T. indicum* group in *T. melanosporum* with RFLP and CGE analysis of the ITS1, 5.8S, and ITS2 regions amplified by ITS1/ITS4 primers were performed. As, for example, shown by Roux et al. (1999) [49] and Paolocci et al. (1997) [32], a differentiation between various *Tuber* species via RFLP is possible. But according to our knowledge, this is the first approach to use this technique for a quantitation of possible admixtures from Asian black truffles in *T. melanosporum* via CGE.

It is known from the literature that genetic differences exist in the ITS region of *Tuber* species, especially in *T. aestivum*. Compared to *T. aestivum,* the other analyzed *Tuber* species show a relatively low intraspecific divergence [48,50]. 

In 2018, Qiao et al. [50] sequenced and analyzed a 500 bp long fragment of the ITS region from 476 truffle ascocarps of the *T. indicum* complex and revealed 54 haplotypes. In the scope of this work, we compared the 476 published ITS sequences with each other, and the restriction site from the endonuclease *Cvi*QI was examined. The sequences can basically be divided into three groups. (i) The first group with one restriction side after base number 30 includes 258 ascocarps, the majority of analyzed samples, (ii) the second group with two restriction sides after base number 60 and 336, 64 and 340 or 60 and 337 includes 203 ascocarps. (iii) The third group, including 15 ascocarps, just a minority of samples shows no restriction sides. In this work, 25 ascocarps of Asian black truffles were analyzed with RFLP from the ITS region. All fruiting bodies but one showed the same restriction pattern with two bands (150 and 500 bp). To compare the obtained restriction profile with the sequences published by Qiao et al. (2018) [50], some ITS amplicons were sequenced (Sanger sequencing, Eurofins Genomics GmbH, Ebersberg, Germany) showing that they are similar to the first group with one restriction side after base number 30. The fruiting body showing a divergent restriction pattern (see Figure S6) belongs to one ascocarp collected in Yunnan, and the sequence comparisons showed that the obtained sequence is similar to the second group with two restriction sides.

The 20 fruiting bodies from *T. melanosporum* analyzed via RFLP showed a uniform species-specific restriction pattern (see Figure S5), indicating that this should not hinder a differentiation of *T. melanosporum* and *T. indicum*.

**Figure 3:**
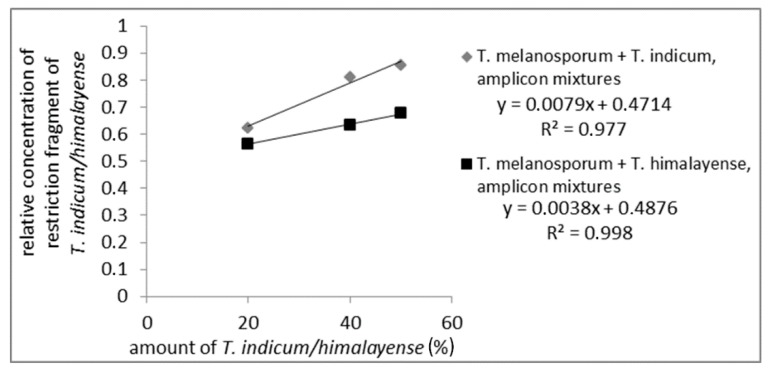
Standard curve of PCR-amplicon mixtures digested with *Cvi*QI from *T. melanosporum* with Asian black truffles added to 50%, 40%, and 20%. The concentration of the long restriction fragment of Asian truffles (500 bp) relative to the total concentration of restriction fragments is plotted against Asian truffle amount.

To check if quantitative assays for a quantitation of admixtures from the Asian black truffles in *T. melanosporum* were possible mixtures of PCR amplicons from *T. melanosporum* with 20%, 40%, and 50%, Asian black truffles were prepared and incubated with the restriction enzyme *Cvi*QI. For the mixtures, samples from the Asian black truffles showing one restriction side were used. The restriction fragments were analyzed via AGE (data not shown) and CGE. The CGE-chromatograms from *T. melanosporum* and *T. indicum/himalayense* measured separately are shown in Figure S7. For CGE evaluation, the long restriction fragments (*T. indicum/himalayense*: 500 bp; *T. melanosporum*: 430 bp) were used. Figure 3 presents the results of the quantitative evaluation, which demonstrate a linear relationship between the Asian black truffle content and the relative amount of characteristic restriction fragments (measuring values are shown in Table S4). It is important to note that a linear correlation could only be observed when the concentration of the characteristic restriction fragment was brought into relation with the total concentration of detected restriction fragments. Otherwise, no linear correlation could be observed. So the total concentration of detected restriction fragments and variations in the measured sample volume were considered. 

**Figure 4:**
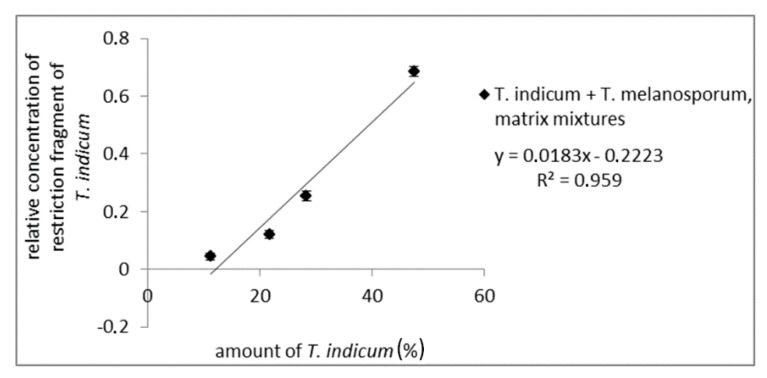
Standard curve of matrix mixtures from fruiting bodies of *T. melanosporum* with Asian black truffles with 11.2%, 21.7%, 28.3%, 47.5% Asian truffle. The concentration of the long restriction fragment (ITS1/ITS4 PCR amplicon digested with *Cvi*QI) of Asian truffles (500 bp) relative to the total concentration of restriction fragments is plotted against Asian truffle amount.

To test the influences of the truffle matrix on the quantitative evaluation mixtures of fruiting bodies from *T. melanosporum,* different amounts of *T. indicum* were prepared. After DNA-isolation, PCR-amplification with the ITS1/ITS4 primer pair, and digestion with *Cvi*QI, PCR fragments were analyzed via AGE (results not shown) and CGE. For each matrix mixture, PCR and enzymatic digestion were performed in a fourfold determination. For a quantitative analysis, the detected concentration of the long restriction fragment relative to the total amount of all detected fragments was plotted against the amount of Asian truffle. As can be seen from Figure 4, the relative peak intensity correlates with the *T. indicum* amount (*R*^2^ of 0.959), demonstrating a possible quantitative determination of possible admixtures with Asian black truffles in *T. melanosporum* samples (measuring values are shown in Table S5). This was comparable with the results of PCR amplicon mixtures. The obtained results show that the CGE assay can be used to determine the amount of admixtures from Asian black truffles in *T. melanosporum,* e.g., for quality control to ensure the integrity of truffle products.

## 4. Conclusions

The results achieved in the present work show that the developed real-time PCR assay with species-specific primer and the CGE-methods allows the identification of different commercially relevant truffle species. The applied real-time PCR is suited to detect and quantify admixtures from Asian black truffles in *T. melanosporum* up to 0.5%. According to our best knowledge, there is no publication to quantify possible admixtures of *T. indicum* in *T. melanosporum* neither with real-time PCR nor with other molecular biological methods. The developed CGE-method based on the ITS region—with and without restriction digestion—offers a promising alternative to real-time PCR. The molecular biological methods developed can be used for quality control in the food industry or in official food control to ensure the integrity of truffle products. 

## Figures and Tables

**Table 1:** Sample material used in this study.

Tuber Species	Geographical Origin	Fruiting Bodies Analyzed	
		Numbers with Regard to the Origin	Total Number
*T. albidum* Pico	Italy	5	5
*T. indicum*	China	5	5
*T. himalayense*	Dali, Yunnan, China	20	20
*T. brumale*	Sarrion, Teruel, Spain	2	2
*T. melanosporum*	Marche, Italy	2	
	France	1	
	Australia	2	
	Sarrion, Teruel, Spain	8	
	Castello, Valencia, Spain	6	
	unknown	1	20
*T. magnatum*	Romagna, Italy	2	
	Buzet, Croatia	1	
	Turin, Piemonte, Italy	1	
	Italy	5	
	L’Aquila, Abruzzo, Italy	1	
	Perugia, Umbria, Italy	1	
	Rome, Lazio, Italy	1	
	Naples, Campania, Italy	1	
	Ancona, Marche, Italy	1	
	Campobasso, Molise, Italy	1	15
*T. aestivum*	unknown	19	
	Romania	15	
	Italy	11	
	Hungary	3	
	Toscana, Florence, Italy	2	50
Processed food containing truffle:			
*T. melanosporum* fruiting bodies canned in saltwater			6
salt with dried *T. aestivum*			1
*T. brumale* chopped and cooked in sherry port wine stock			1

**Table 2:** Primer and probe sequences and size of PCR products.

Primer/Probes	Name	Sequence 5′–3′	Product Size (bp)
specific for *T. melanosporum*	Primer Mela-fw Mela-rv Probe	ACGACGGACTTTATAAACGGTTATAA AGCGGGTATCCCTCCCTGATT Cy5–GACCTGGATCAGTCACAAGTCTTGTCTGGT-BHQ2	141
specific for *T. indicum/* *T. himalayense*	Primer Indi-fw ITS4LNG * Probe	AACAACAGACTTTGTAAAGGGTT TGATATGCTTAAGTTCAGCGGG HEX-GGACCTAGATCAGTCACAAGTCATGTCTGG-BHQ2	146

fw = forward; rv = reverse, * Paolocci, et al. (1997) [32]

## Supplementary Materials

The following are available online at https://www.mdpi.com/2304-8158/9/4/501/s1, Table S1: Results of the specificity test of the specific real-time primers, Table S2: Measuring values that were used as the basis for preparing the standard curves for Figures 1, Table S3: Measuring values that were used as the basis for preparing the standard curves for Figures 2, Table S4: Measuring values that were used as the basis for preparing the standard curves for Figures 3, Table S5: Measuring values that were used as the basis for preparing the standard curves for Figures 4, Figure S1: Results from the amplification with the ITS1/4 primer pair, DNA isolated of *T. magnatum* fruiting bodies, Figure S2: Results from the amplification with the ITS1/4 primer pair, DNA isolated of *T. albidum* Pico fruiting bodies, Figure S3: Results from the amplification with the ITS1/4 primer pair, DNA isolated of *T. aestivum* fruiting bodies, Figure S4: Results from the amplification with the ITS1/4 primer pair, DNA isolated of *T. brumale* fruiting bodies and food truffle products, Figure S5: Results from the amplification with the ITS1/4 primer pair and the RFLP assay with CviQI, DNA isolated of *T. melanosporum* fruiting bodies, Figure S6: Results from the amplification with the ITS1/4 primer pair and the RFLP assay with CviQI, DNA isolated from *T. indicum/himalayense* fruiting bodies, Figure S7: CGE-chromatogram, PCR-fragments generated with ITS1/ITS4 primer pair after digestion with CviQI, (A) *T. indicum* DNA: 130 bp, 498 bp; (B) *T. melanosporum*: 193 bp, 438 bp.
