# Supplementary material for: Food Authentication: Identification and Quantitation of Different Tuber Species via Capillary Gel Electrophoresis and Real-Time PCR
Source: Foods. 2020 Apr 16;9(4):501. doi: 10.3390/foods9040501 (PMC7230311; doi:10.3390/foods9040501)
Supplement: Supplementary file 1 [file foods-09-00501-s001.pdf]

Food Authentication: Identification and quantitation of different Tuber species via capillary gel electrophoresis and real-time PCR

Stefanie Schelm [1], Melanie Siemt [1], Janin Pfeiffer [1], Christina Lang [1], Hans-Volker Tichy [2] and Markus Fischer [1,*]

[1] HAMBURG SCHOOL OF FOOD SCIENCE; Institute of Food Chemistry, University of Hamburg, Grindelallee 117, 20146 Hamburg, Germany; stefanie.schelm@chemie.uni-hamburg.de, melanie.siemt@studium.uni-hamburg.de, janin.pfeiffer@studium.uni-hamburg.de, christina.lang@chemie.uni-hamburg.de

[2] LUFA-ITL GmbH, Dr.-Hell-Straße 6, 24107 Kiel, Germany; hans-volker.tichy@agrolab.de

* Correspondence: Markus.Fischer@uni-hamburg.de; Tel.: Tel.: +49-40-428384357

Table S1. Results (Cq values) of specificity test with the *T. indicum* (Indi-fw/ ITS4LNG) and the *T. melanosporum* specific primer pair (Mela-fw/Mela-rv) in the real-time PCR assay. DNA isolated from different *Tuber* spp. ascocarps showing positive results with *T. indicum* and *T. himalayense* DNA using the *T. indicum* specific primer pair and with *T. melanosporum* using the *T. melanosporum* specific primer pair.

Number of Fruiting Body Analyzed	Geographical Origin	Cq Value *T. melanosporum* Specific Primer	Cq Value *T. indicum* Specific Primer
***T. albidum* Pico (total: 5)**			
1 - 5	Italy	N/A	N/A
***T. indicum* (total: 5)/ *T. himalayense* (total: 20)**			
1	China (*T. indicum*)	N/A	25.45
2		N/A	23.43
3		N/A	25.03
4		N/A	19.43
5		N/A	21.00
1	Dali, Yunnan, China (*T. himalayense*)	N/A	19.12
2		N/A	14.70
3		N/A	20.03
4		N/A	20.54
5		N/A	23.13
6		N/A	14.89
7		N/A	21.68
8		N/A	23.02
9		N/A	18.08
10		N/A	21.92
11		N/A	19.82
12		N/A	21.16
13		N/A	20.02
14		N/A	19.28
15		N/A	15.93
16		N/A	19.92
17		N/A	22.12
18		N/A	18.35
19		N/A	18.85
20		N/A	15.72
***T. brumale* (total: 2)**			
1 - 2	Sarrion, Teruel, Spain	N/A	N/A

Table S1. continued.

Number of Fruiting Body Analyzed	Geographical Origin	Cq Value *T. melanosporum* Specific Primer	Cq Value *T. indicum* Specific Primer
	T. melanosporum **(**total: 20)		
1	Marche, Italy	22.19	N/A
2		22.29	N/A
1	France	21.09	N/A
1	Australia	21.46	N/A
2		19.69	N/A
1	Sarrion, Teruel, Spain	15.67	N/A
2		16.67	N/A
3		17.43	N/A
4		20.42	N/A
5		16.06	N/A
6		18.94	N/A
7		23.8	N/A
8		16.08	N/A
1	Castello, Valencia, Spain	15.01	N/A
2		15.48	N/A
3		15.98	N/A
4		15.21	N/A
5		17.03	N/A
6		14.31	N/A
1	unknown	22.98	N/A
	T. magnatum **(**total: 15)		
1–15	Italy, Croatia	N/A	N/A
	T. aestivum **(**total: 50)		
1–50	unknown, Italy, Romania, Hungary	N/A	N/A
	**processed food truffle products from food retailing**		
1	*T. melanosporum* fruiting bodies canned in saltwater	29.48	N/A
2		24.17	N/A
3		29.54	N/A
4		25.15	N/A
5		20.12	N/A
6		21.83	N/A
1	salt with dried *T. aestivum*	N/A	N/A
1	*T. brumale* chopped and cooked in sherry port wine stock	N/A	N/A

Table S2. Measuring values that were used as the basis for preparing the standard curves for Figures 1.

Standard Curve of Real-Time PCR of DNA-mixtures from T. *melanosporum* with *T. indicum*					
**Measure ment**	**Amount of *T. indicum* [%]**	**Logarithm of the Amount of *T. indicum***	**Cq Value**	**Mean of Cq Values**	**Standard Deviation of Cq Values**
1	0.50	-0.30	28.66	28.85	0.26
2			29.03		
1	1.00	0.00	27.31	27.16	0.21
2			27.01		
1	5.00	0.70	24.73	24.32	0.58
2			23.91		
1	10.00	1.00	23.28	23.22	0.09
2			23.15		
1	20.00	1.30	22.01	22.11	0.13
2			22.20		
1	40.00	1.60	20.77	20.97	0.28
2			21.17		
1	70.00	1.85	21.22	20.48	1.05
2			19.73		
**Standard curve of real-time PCR of matrix-mixtures from T. *melanosporum* with *T. indicum***					
**measure-ment**	**amount of *T. indicum* [%]**	**logarithm of the amount of *T. indicum***	**Cq value**	**mean of Cq values**	**standard deviation of Cq values**
1	4.30	0.63	27.5	27.33	0.25
2			27.05		
3			27.45		
1	7.40	0.87	23.77	24.25	0.70
2			25.05		
3			23.94		
1	13.50	1.13	24.05	23.73	0.34
2			23.78		
3			23.37		
1	20.40	1.31	22.58	21.88	1.30
2			20.38		
3			22.68		
1	32.17	1.51	19.83	20.65	0,82
2			21.46		
3			20.67		

Table S3. Measuring values that were used as the basis for preparing the standard curves for Figures 2.

Standard Curve of PCR-amplicon Mixtures from *T. indicum* with *T. aestivum*			
**Amount of *T. indicum* [%]**	**Area of PCR-Amplicons from *T. indicum* [nmol]**	**Total Area of PCR-amplicons [nmol]**	**Relative Area of PCR-amplicons from *T. indicum***
5.00	2.20	37.60	0.06
20.00	5.10	24.70	0.21
40.00	9.60	23.60	0.41
80.00	19.90	24.10	0.83

Standard curve of PCR-amplicon mixtures from *T. albidum* with *T. magnatum*					
**mea sure - men t**	**amount of *T. albidum* [%]**	**area of PCR-amplicons from *T. albidum* [nmol]**	**total area of PCR-amplicons [nmol]**	**relative area of PCR-amplicons from *T. albidum***	**mean of relative areas of PCR-amplicons from *T. albidum***
1	5.00	18.70	38.30	0.49	0.48
2		9.70	20.90	0.46	
Table S3 nicht ganz klar. 1	20.00	27.80	39.70	0.70	0.70
2		16.00	22.60	0.71	
1	40.00	39.20	45.40	0.86	0.84
2		18.60	22.80	0.82	
1	80.00	48.10	50.10	0.96	0.96
2		28.90	29.80	0.97	

Standard curve of DNA-amplicon mixtures from *T. albidum* with *T. magnatum*			
**amount of *T. albidum* [%]**	**area of PCR-amplicons from *T. albidum* [nmol]**	**total area of PCR-amplicons [nmol]**	**relative area of PCR-amplicons from *T. albidum***
5.00	0.40	6.70	0.06
20.00	0.90	6.20	0.15
40.00	1.70	5.70	0.30
80.00	3.50	4.80	0.73

Table S4: Measuring values that were used as the basis for preparing the standard curves for Figures 3.

**Standard Curve of PCR-amplicon Mixtures Digested with *Cvi*QI from *T. melanosporum* with *T. indicum***			
**Amount of *T. indicum* [%]**	**concentration of the long restriction fragment from *T. indicum* [ng/µL]**	**total concentration of the long restriction fragments [ng/µL]**	**Relative Concentration of The Long Restriction Fragment of *T. indicum***
20.00	0.96	1.54	0.62
40.00	1.71	2.11	0.81
50.00	5.40	6.32	0.85
**Standard curve of PCR-amplicon mixtures digested with *Cvi*QI from *T. melanosporum* with *T. himalayense***			
**amount of *T. himalayense* [%]**	**concentration of the long restriction fragment from *T. himalayense* [ng/µL]**	**total concentration of the long restriction fragments [ng/µL]**	**relative concentration of the long restriction fragment of *T. himalayense***
20.00	0.22	0.39	0.56
40.00	0.47	0.74	0.64
50.00	0.58	0.87	0.68

Table S5: Measuring values that were used as the basis for preparing the standard curves for Figures 4.

**Standard Curve of Matrix Mixtures from Fruiting Bodies of *T. Melanosporum* with Asian Black Truffles**						
**Measurement**	**Amount of *T. himala-yense* [%]**	**Concentration of the Long Restriction Fragment from *T. himalayense* [ng/µL]**	**Total Concentra-Tion of the Long Restriction frag-ments [ng/µL]**	**Relative Concentra-Tion of the Long Restriction Fragment of *T. himalayense***	**Mean of Cq Values**	**Standard Deviation of Cq Values**
1	11.18	0.43	11.29	0.04	0.04	0.01
2		0.18	4.26	0.04		
3		0.03	0.81	0.04		
4		0.20	3.21	0.06		
1	21.70	0.44	3.97	0.11	0.12	0.01
2		0.62	4.42	0.14		
3		0.26	2.16	0.12		
4		0.15	1.38	0.11		
1	28.29	0.58	3.75	0.23	0.25	0.02
2		0.84	3.22	0.26		
3		1.17	4.47	0.26		
4		0.33	1.26	0.26		
1	47.51	2.67	3.92	0.68	0.68	0.02
2		0.16	0.16	0.66		
3		2.11	3.15	0.67		
4		1.16	1.65	0.70		

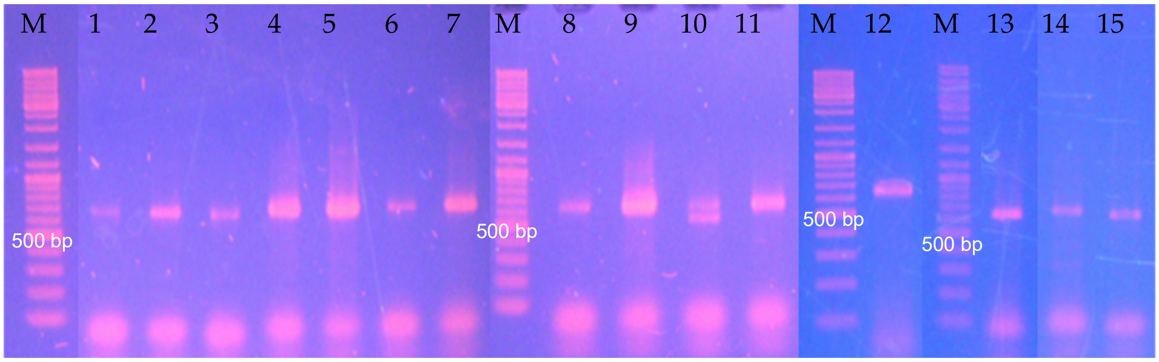

Figure S1. Results from the amplification with the ITS1/4 primer pair, DNA isolated of *T. magnatum* fruiting bodies; M=marker. Geographical origin of the samples with the corresponding allocations on the agarose gel: 1, Croatia, Buzet; 2, Italy, Piedmont, Turin; 3-5, Italy; 6, Italy, Abruzzo, L'Aquila; 7, Italy, Umbria, Perugia; 8, Italy, Latium, Rome; 9, Italy, Campania, Naples; 10, Italy, Marche, Ancona; 11, Italy, Molise, Campobasso; 12-13, Italy, Romagna¸14-15, Italy.

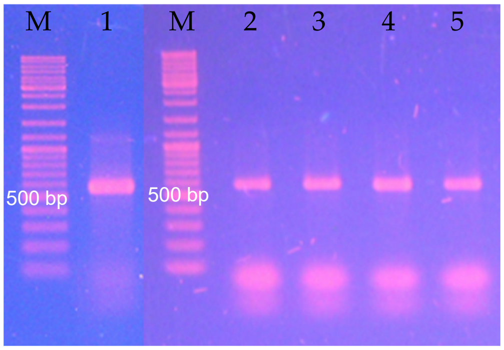

Figure S2: Results from the amplification with the ITS1/4 primer pair, DNA isolated of *T. albidum* Pico fruiting bodies; M=marker. Geographical origin of the samples with the corresponding allocations on the agarose gel: 1-5, Italy.

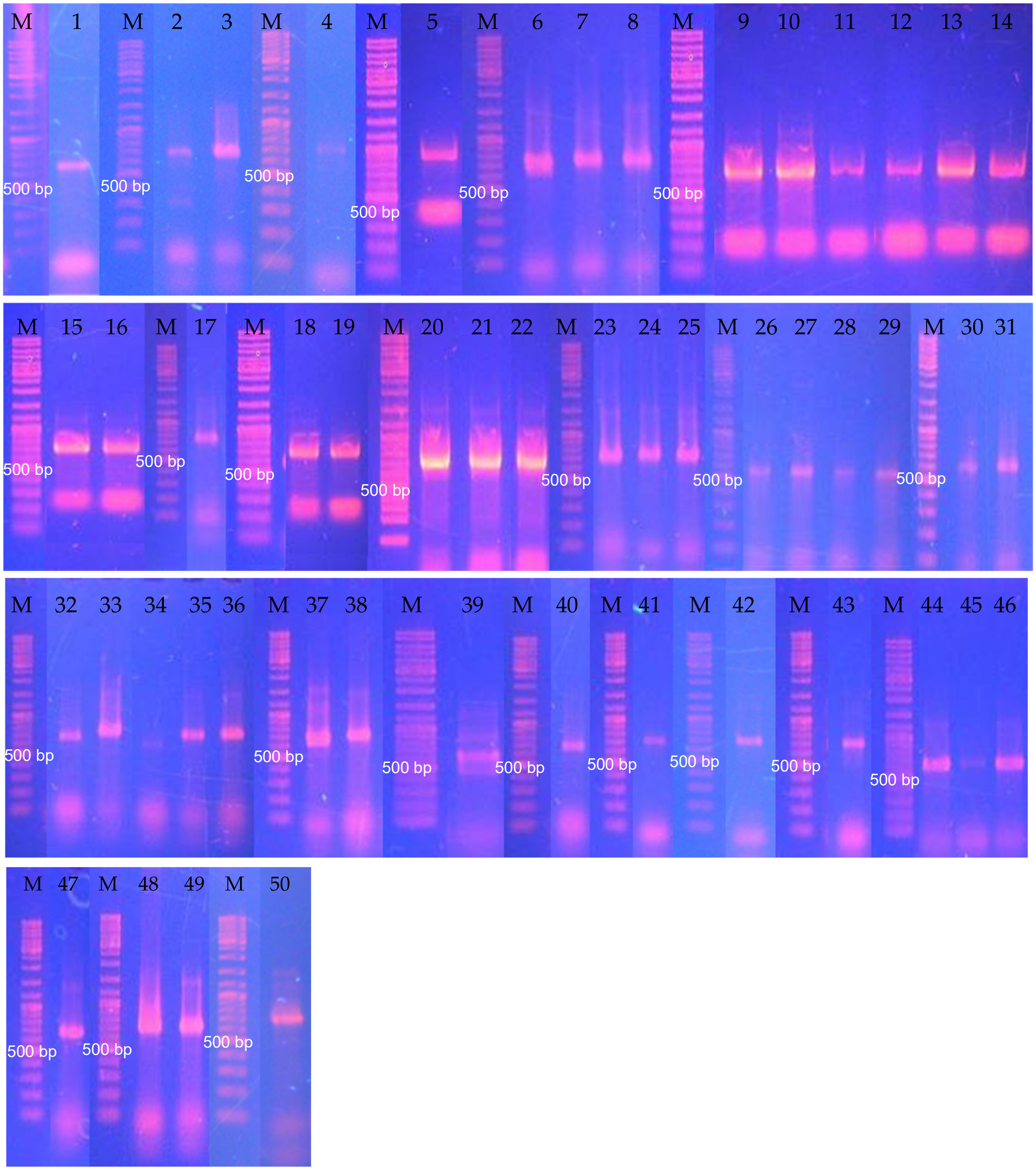

Figure S3: Results from the amplification with the ITS1/4 primer pair, DNA isolated of *T. aestivum* fruiting bodies; M=marker. Geographical origin of the samples with the corresponding allocations on the agarose gel: 1-3, Hungary; 4-13, unknown; 14-16, Italy; 17-18, Italy, Tuscany, Florence; 19-25, Italy; 26-40, Romania; 41-49, unknown; 32, Italy.

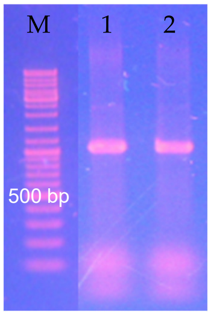

Figure S4: Results from the amplification with the ITS1/4 primer pair, DNA isolated of *T. brumale* fruiting bodies and food truffle products; M=marker. Geographical origin of the samples with the corresponding allocations on the agarose gel: 1-2, Spain, Teruel, Sarrion.

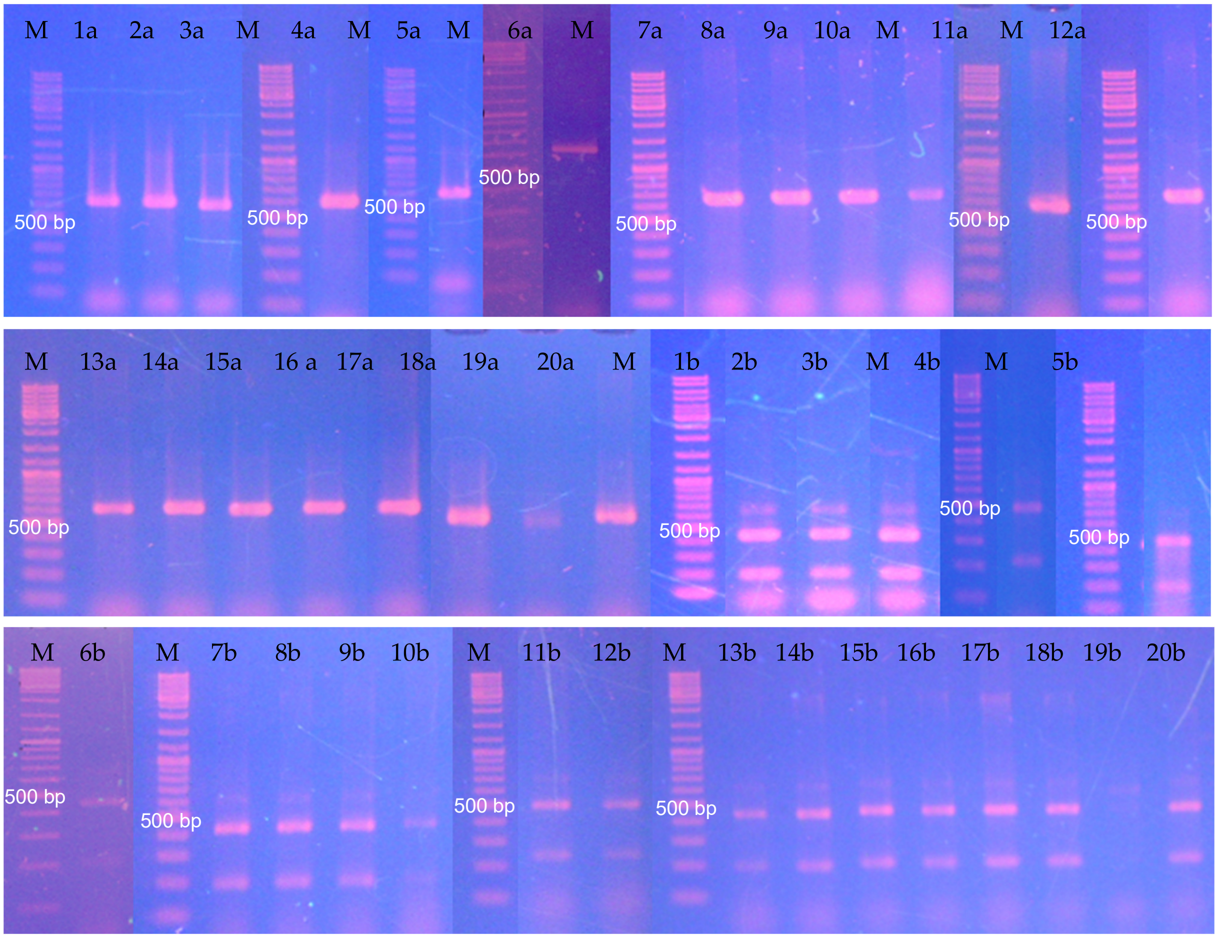

Figure S5: Results from the amplification with the ITS1/4 primer pair and the RFLP assay with CviQI, DNA isolated of *T. melanosporum* fruiting bodies; a: PCR amplicons bevor restriction, b: amplicons after restriction; M=marker. Geographical origin of the samples with the corresponding allocations on the agarose gel: 1-2, Australia; 3, France; 4-5, Italy, Marken; 6, unknown; 7-12, Spain, Valencia, Castello, 13-20, Spain, Teruel, Sarrion.

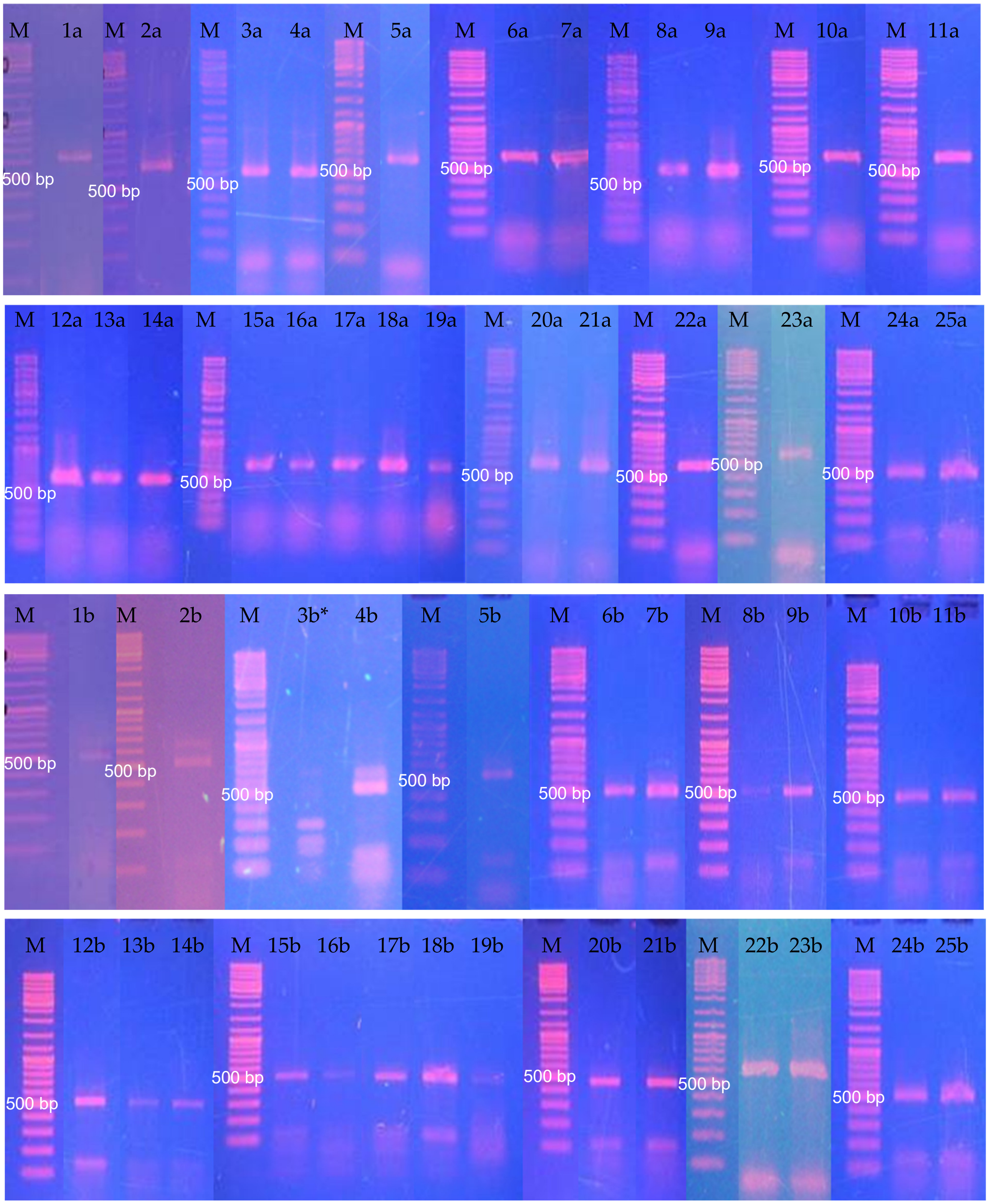

Figure S6: Results from the amplification with the ITS1/4 primer pair and the RFLP assay with CviQI, DNA isolated of *T. indicum/ himalayense* fruiting bodies; a: PCR amplicons bevor restriction, b: amplicons after restriction, M=marker. Geographical origin of the samples with the corresponding allocations on the agarose gel: 1-5, China; 5-25, China, Yunnan, Dali.

*divergent restriction pattern

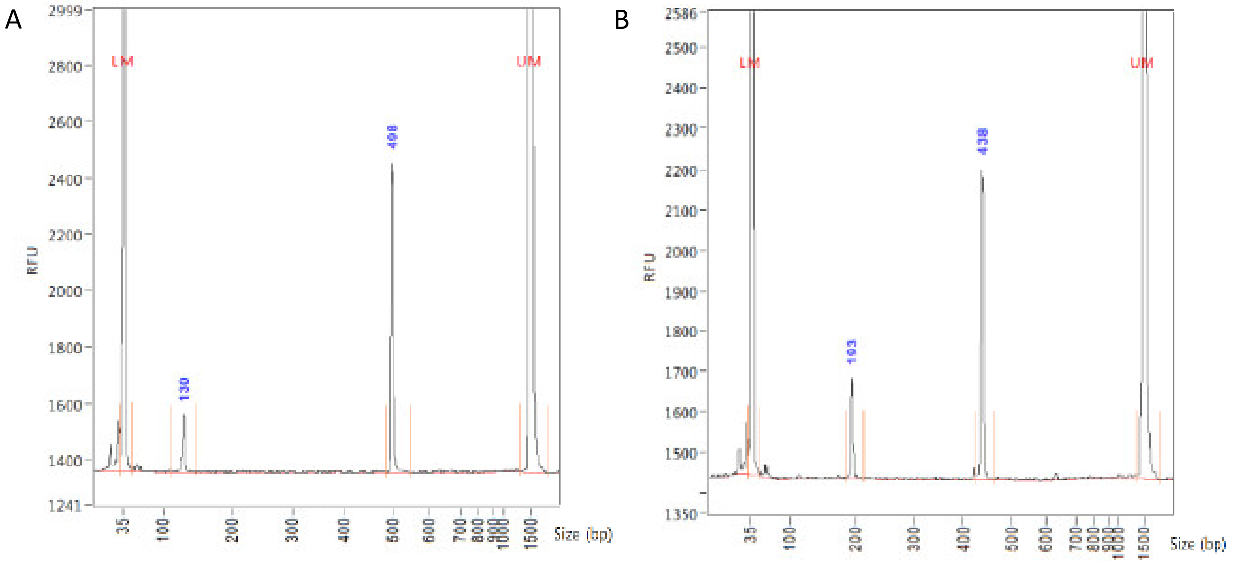

Figure S7: CGE-chromatogram, PCR-fragments generated with ITS1/ITS4 primer pair after digestion with CviQI, A) *T. indicum* DNA: 130 bp, 498 bp; B) *T. melanosporum*: 193 bp, 438 bp, LM: Lower Marker (35 bp), UP: Upper Marker (1500 bp).

Equitation for the correlation coefficient R^2:

$$R^2 = \left(\frac{\Sigma(x - \overline{y})(y - \overline{y})}{\sqrt{\Sigma(x - \overline{x})^2 \sqrt{(y - \overline{y})^2}}} \right)^2 \tag{S1}$$
